# Silence of NLRP3 Suppresses Atherosclerosis and Stabilizes Plaques in Apolipoprotein E-Deficient Mice 

**DOI:** 10.1155/2014/507208

**Published:** 2014-06-04

**Authors:** Fei Zheng, Shanshan Xing, Zushun Gong, Wei Mu, Qichong Xing

**Affiliations:** ^1^Department of Cardiology, Qianfoshan Hospital, Shandong University, 16766 Jingshi Road, Jinan 250014, China; ^2^Shandong University of Traditional Chinese Medicine, Jinan 250355, China

## Abstract

*Objectives*. The role of the NLRP3 inflammasome in atherosclerosis remains controversial. The aim of this study was to determine whether inhibition of NLRP3 signaling by lentivirus-mediated RNA interference could reduce atherosclerosis and stabilizes plaques. We also tried to explore the mechanisms of the impact of NLRP3 inflammasome on atherosclerosis. *Methods*. Apolipoprotein E-deficient mice aged 8 weeks were fed a high-fat diet and were injected with NLRP3 interfering or mock viral suspension after 4 weeks. Lentivirus transfer was repeated in 2 weeks. Four weeks after the first lentivirus injection, we evaluated the effects of NLRP3 gene silencing on plaque composition and stability and on cholesterol efflux and collagen metabolism, by histopathologic analyses and real-time PCR. *Results*. Gene silence of NLRP3 prevented plaques progression and inhibited inductions of proinflammatory cytokines. Moreover, this RNA interference reduced plaque content of macrophages and lipid, and increased plaque content of smooth muscle cells and collagen, leading to the stabilizing of atherosclerotic plaques. *Conclusions*. NLRP3 inflammasomes may play a vital role in atherosclerosis, and lentivirus-mediated NLRP3 silencing would be a new strategy to inhibit plaques progression and to reduce local inflammation.

## 1. Introduction

Atherosclerosis is a complicated inflammatory progress characterized by lipid deposition, leukocyte infiltration, and vascular smooth muscle cell proliferation in the vascular walls [1, 2]. Because inflammation in the atherosclerotic process mostly occurs in the absence of microbial infection, it is considered to be sterile inflammation [3]. Growing evidences suggest that some types of sterile inflammation are mediated by the inflammasome, a large multiprotein complex in the cytosol and a component of the innate immune system [4, 5]. The nod-like receptor family pyrin domain containing 3 (NLRP3) inflammasome, the best characterized inflammasome to date, contains the nod-like receptor scaffold protein NLRP3 associated with the adapter protein, apoptosis-associated speck-like protein containing a CARD (ASC), which recruits caspase-1 and induces its activation. Caspase-1 induces secretion of interleukin-1*β* (IL-1*β*) and interleukin-18 (IL-18), which plays a vital role in promoting the development of lipid plaques and destabilizing the plaques [6, 7]. These findings suggest the involvement of the NLRP3 inflammasome in the development of atherosclerosis.

Recent studies have shown that cholesterol crystal activate the NLRP3 inflammasome and release of IL-1*β*, which indicates that the NLRP3 inflammasome may have a role in the development of atherosclerosis [8, 9]. However, another study of double-mutant mice did not find any differences in atherosclerosis progression with or without the NLRP3 inflammasome [10]. The reason for this discrepancy is unclear. The role of the NLRP3 inflammasome in atherosclerosis remains controversial.

Small interfering RNAs (siRNAs) have proven effective in silencing target genes and lentivirus can efficiently deliver siRNA [11]. In the present study, we constructed lentiviral vectors to knock down NLRP3 to evaluate the role of the NLRP3 inflammasome in atherosclerosis in apolipoprotein (Apo) E-deficient mice, which can develop severe hypercholesterolemia and spontaneous atherosclerosis [12].

## 2. Materials and Methods

### 2.1. Lentivirus Vectors for NLRP3 RNAi

Three different NLRP3-specific target sequences were chosen using the NLRP3 reference sequence (Gene Bank Accession number NC_000077). According to the structure of a pGCSIL-GFP viral vector (Genechem gene, Shanghai, China), double-stranded DNA were synthesized and inserted into a linearized vector. The positive clones were identified as lentiviral vectors and pGCSIL/NLRP3-1 (sequence: 5′-GGUGAAAUGUACUUAAAUC-3′) induced the highest levels of downregulation. So pGCSIL/NLRP3-1 vector and viral packaging system were cotransfected into 293 cells to replicate competent lentivirus. The mock vector (sequence: 5′-GUGCACAUGAGUGAGAUUU-3′) was also packaged and used as a negative control, which has no significant homology to mouse gene sequences.

### 2.2. Animal Protocol

The animal experimental protocol was reviewed and approved by the Animal Care Committee of Shandong University (Jinan, China). A total of 40 male Apo E-deficient mice aged 8 weeks were purchased from the Beijing University Animal Research Center (Beijing, China) and were housed on a 12-hr light/12-hr dark cycle in plenty of food and water. All mice were fed a high-fat diet (0.25% cholesterol and 15% cocoa butter). After 4 weeks, mice were divided into control group and NLRP3 interfering (NLRP3i) group, which were injected via tail vein with mock viral suspension (1.75 × 10^8^ Tfu, 20 uL) and NLRP3i viral suspension (1.75 × 10^8^ Tfu, 20 uL). Lentivirus transfer was repeated in 2 weeks. Four weeks after the first lentivirus injection, mice were anesthetized and perfused with phosphate buffer saline (PBS) for 2 min with needle inserted into the left ventricle. The aortic root vessels were removed and fixed in 4% paraformaldehyde overnight and then embedded in optimal cutting temperature (OCT) compound. The rest of aortas were collected for protein extraction and PCR.

### 2.3. Serum Lipid Level Measurement

Total triglycerides and cholesterol were detected with an automated enzymatic technique (Boehringer Mannheim Diagnostics) [13]. High-density lipoprotein (HDL) and low-density lipoprotein (LDL) levels were determined with an automated chemically modified technique (Roche Modular DPP System, Roche, Switzerland).

### 2.4. Caspase-1 Activity Measurement and ELISA

Caspase-1 activity in protein extracted from aortas in the control and NLRP3i groups was measured using the Caspase-1 Colorimetric Assay Kit (BioVision, Mountain View, CA) according to the manufacturer's instructions. Absorbance reading was taken at 410 nm using a microplate reader. IL-1*β* and IL-18 were analysed using IL-1*β*- and IL-18-specific ELISA kits from R&D Systems.

### 2.5. Histological Analysis

OCT compound-embedded aortic root vessels were cross-sectioned into pieces 6 *μ*m thick at 50 *μ*m intervals. Sections were stained with hematoxylin and eosin (H&E) for histological analysis. The plaque was subdivided into a fibrous cap and a necrotic core on the basis of picrosirius red staining for collagen and oil red O staining for lipids, respectively. Corresponding sections on separate slides were immunostained with monoclonal antibodies. Macrophages and smooth muscle cells (SMCs) were immunostained with monocyte/macrophage-specific antibody (MOMA)-2 (diluted 1 : 25; Abcam, Cambridge, MA) and anti-*α*-actin antibodies (diluted 1 : 1000; Sigma-Aldrich), respectively. The sections were incubated with the appropriate HRP-conjugated secondary antibodies (diluted 1 : 100; Zhongshan), followed by incubation with 3,3′-diaminobenzidine and counterstaining with hematoxylin. Sections reacted with nonimmune IgG, secondary antibody only, and no primary and secondary antibodies were used as negative controls.

An automated image analysis system (Image-Pro Plus 6.0; Media Cybernetics, Silver Spring, MD) was used for quantitative measurements. The positive-staining area of macrophages, SMCs, lipids, and collagen was quantified by computer-assisted color-gated measurement, and the ratio of positive-staining area to intimal area was calculated. The vulnerability index was calculated by the following formula: positive-staining area of (macrophages + lipid)/positive-staining area of (*α*-SMCs + collagen) [14].

### 2.6. Quantitative Real-Time PCR Analysis

After the aortas were collected, total RNA was extracted using Trizol reagent (Invitrogen) in accordance with the manufacturer's instructions. The mRNA levels were quantified by the use of SYBR green technology. The house-keeping gene *β*-actin was quantified as an internal RNA control. The primer sequences for all studied genes are listed in Table 1. The 2^−ΔΔCT^   method for comparing relative expression results was applied [15].

### 2.7. Data Analysis

Data were analyzed using SPSS 18.0 (SPSS Inc., Chicago, IL, USA). Quantitative variables are presented as mean ± SD. A 2-tailed Student's *t*-test was used in the comparison of continuous data. *P* < 0.05 was considered statistically significant.

## 3. Results

### 3.1. Safety and Efficiency of Lentiviral Transfection

**Figure 1:**
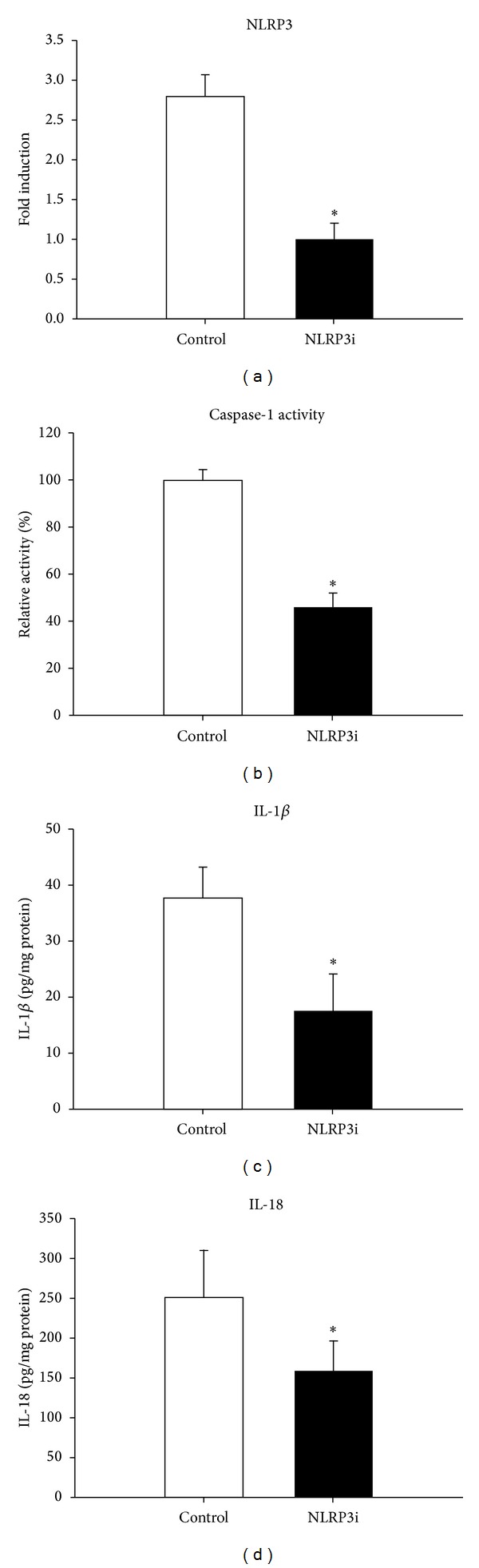
Efficiency of lentivirus transfection. (a) NLRP3 interference downregulated NLRP3 mRNA expression by 64.3% compared with the control via real-time PCR analyses. (b) Caspase-1 activity in protein extracted from aortas was reduced by 56% after NLRP3 silencing, compared with the control. (c and d) Silence of NLRP3 decreased the downstream proinflammatory factors, IL-1*β* and IL-18, using ELISA. *P < 0.05 versus control. Values are mean ± SD.

All mice were apparently healthy, and no mice were lost before the day of sacrifice. A systemic lentivirus delivery has proven an efficient approach to transferring gene into arterial walls [16]. In order to evaluate the efficacy of lentivirus-mediated gene silencing in vivo, the levels of NLRP3 mRNAs expression, the activity of caspase-1, and the protein level of IL-1*β* and IL-18 (downstream targets of NLRP3 inflammasome) were measured. Compared with the control group, the mRNA levels of NLRP3 were significantly downregulated, the caspase-1 activity was strongly decreased, and the content of IL-1*β* and IL-18 was also decreased after silencing NLRP3 (Figure 1, *P*< 0.05).

### 3.2. Effects of NLRP3i on Plaque Composition and Stability

**Figure 2:**
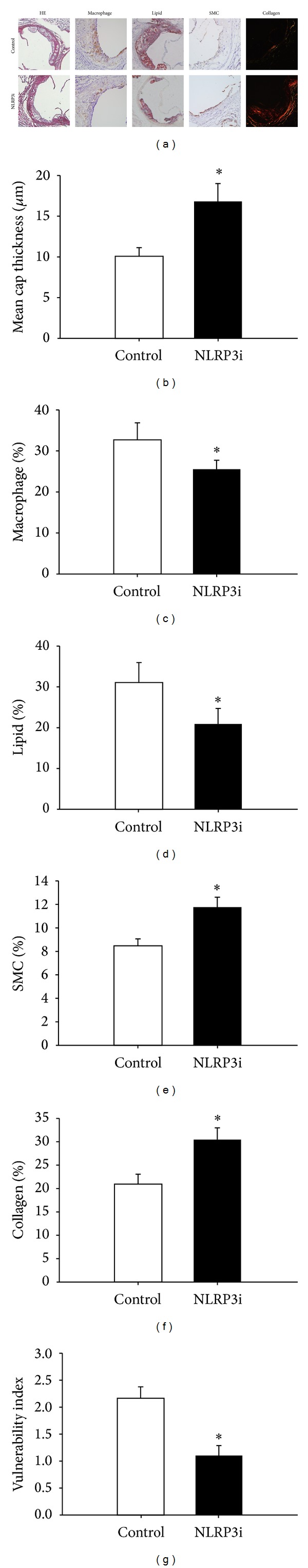
Cross sections of aortic root vessels from the control and gene interference groups were stained with H&E; vascular smooth muscle cells and collagen were immunostained with *α*-actin and Sirius red, respectively; and macrophages and lipids were stained with MOMA-2 and oil red O, respectively (a). The mean cap thickness was increased after the NLRP3 silencing, compared with the control (b). Number of macrophages in the atherosclerotic lesion area was significantly lower by nearly 22% and lipid content was 33% lower with NLRP3 lentivirus than control transfection (c and d). SMCs content was higher by 39% and collagen content was 46% higher with NLRP3 lentivirus than control transfection (e and f). The vulnerability index was significantly lower in NLRP3i group than control (g). *P < 0.05 versus control. Values are mean ± SD. Scale bar is 100 *μ*m.

Mice transfected with control lentivirus or NLRP3 lentivirus differed in histological and immunohistochemical staining of aortic root vessels (Figure 2). In the atherosclerotic lesion area, SMCs content was higher by 39% and collagen content was 46% higher with NLRP3 lentivirus than control transfection. The number of macrophages in the atherosclerotic lesion area, as determined by MOMA-2, was significantly lower by nearly 22% and lipid content was 33% lower with NLRP3 lentivirus than control transfection. Through calculation, the vulnerability index was significantly lower with NLRP3 lentivirus than control transfection.

### 3.3. Effects of NLRP3 Silencing on Body Weight and Lipids

NLRP3 silencing resulted in no significant differences in comparison with controls in body weight. Likewise, serum total cholesterol, triglycerides, LDL, and HDL in the NLRP3i group did not differ significantly from those in the control group (Table 2), suggesting that the effect of gene transfer on atherosclerosis was independent of serum lipid levels.

### 3.4. Effects of NLRP3i on Lipid Efflux

**Figure 3:**
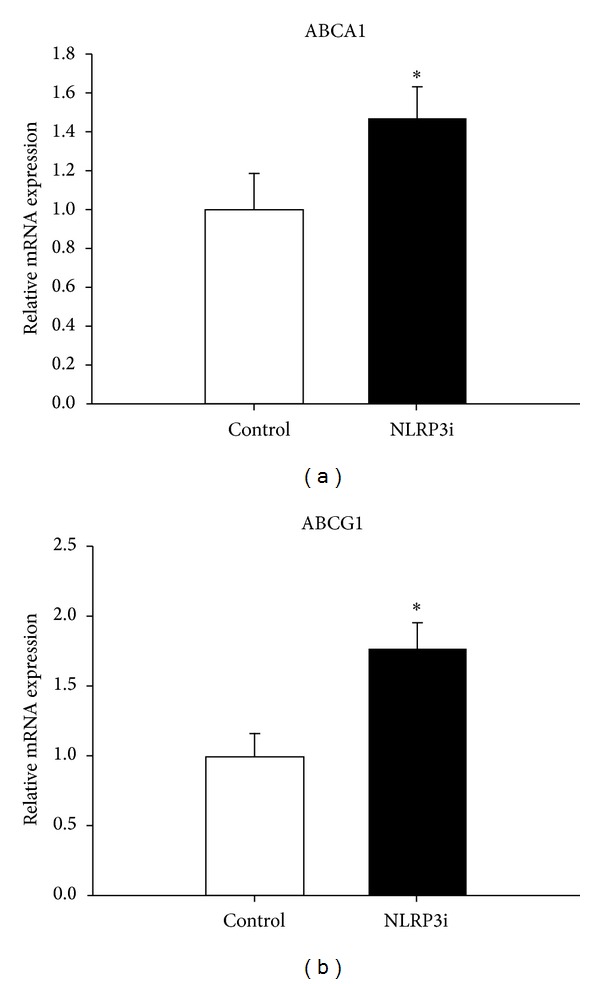
ABCA1 and ABCG1 mRNA expression levels were determined by real-time PCR analyses. Compared with control, mRNA of ABCA1 and ABCG1 increased significantly in aortas of ApoE-deficient mice treated with NLRP3 silencing. *P < 0.05 versus control. Values are mean ± SD.

In order to evaluate the effect of NLRP3 interfering on macrophage lipid efflux, we measured the mRNA expression of ATP-binding cassette (ABC) transporters ABCA1 and ABCG1, also known as the cholesterol efflux regulatory protein. In comparison with control, mRNA of ABCA1 and ABCG1 increased significantly in aortas of ApoE-deficient mice treated with NLRP3 silencing (*P* < 0.05) (Figure 3).

### 3.5. Effects of NLRP3 Silencing on Collagen Synthesis and Degradation

**Figure 4:**
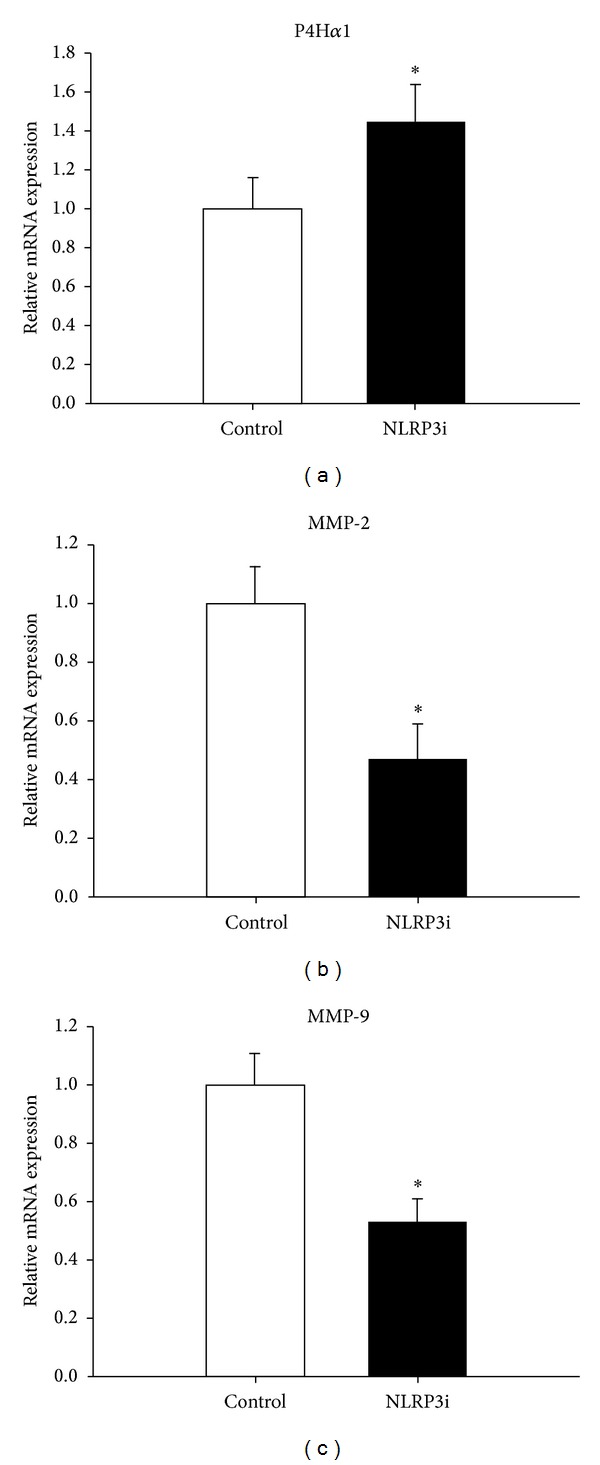
P4H*α*1, MMP-2, and MMP-9 mRNA levels in aortas of ApoE-deficient mice were determined with real-time PCR. The mRNA expression of P4H*α*1 was markedly higher in the NRLP3i group than in the control group, and the mRNA expression levels of MMP-2 and MMP-9 were significantly lower in the treatment group than the control group. *P < 0.05 versus control. Values are mean ± SD.

Collagens are main components of fibrous cap in plaques, and prolyl-4-hydroxylases *α*1 (P4H*α*1) are rate limiting for collagen synthesis. On the contrary, matrix metalloproteinase-2 (MMP-2) and matrix metalloproteinase-9 (MMP-9) contribute to the breakdown of the collagen. Our data showed that the mRNA expression of P4H*α*1 was markedly higher in the NRLP3i group than in the control group (*P* < 0.05), and the mRNA expression of MMP-2 and MMP-9 was significantly lower in the treatment group than the control group (*P* < 0.05) (Figure 4). These two effects led to the increasing of the collagen and thickness of the fibrous cap.

## 4. Discussion

In the present study, we applied lentivirus-mediated RNA interfering methods to efficiently knock down NLRP3 gene and investigated its role in the pathogenesis of plaque vulnerability in ApoE-deficient mice. Our results demonstrated that mRNA expression of NLRP3 was remarkably attenuated by transfected lentiviral siRNAs. Plaques of the NLRP3i group showed a higher content of collagen and SMCs, lower content of lipid and macrophages, and lower vulnerability index than did in the control group.

RNA interference is a powerful technique for selectively silencing mRNA for a wide range of proteins [17], and using siRNA is also more efficient than other gene-specific targeting approaches [18]. Furthermore, gene silencing strategies using siRNA have been applied to treat or prevent several diseases [19, 20]. The major limitation for synthetic siRNAs and plasmid-based siRNAs as a delivering approach is their transient expression and poor transfection efficiency in vivo. In contrast, the lentiviral vector-mediated gene delivery system applied in this study has proven effective and long lasting in silencing gene function in primary mammalian cells, stem cells, and transgenic mice [21]. In the present study, the efficacy of this method was confirmed not only by our observation that NLRP3 mRNA expression levels of the treatment group were remarkably downregulated, but also by observation that the caspase-1 activity was strongly reduced and the content of downstream targets of NLRP3 inflammasomes, IL-1*β* and IL-18, was decreased after silencing NLRP3. In addition, the fact that no adverse effects were observed in our mice during the experiment demonstrated that lentivirus transfection was safe in these animals. Thus, we regard that lentiviral vectors expressing siRNA provide an efficient, specific, and safe tool to knock down NLPR3 genes.

A strong link between inflammation and atherosclerosis is becoming increasingly evident [22]. A number of recent landmark studies have implicated the activation of the NLRP3 inflammasome, in the progress of atherosclerosis. Cholesterol crystals generated in vitro activate caspase-1 and the cleavage of both IL-1*β* and IL-18 in human peripheral blood mononuclear cells and mouse macrophages, and both depend on NLRP3 and ASC [8, 9]. In line with the in vitro data, hypercholesterolemic mice deficient in the receptor for low-density lipoprotein (LDL) which are reconstituted with bone marrow from* Nlrp*3^−/−^,* Pycard* ^−/−^, or* Il1b* ^−/−^ mice develop many fewer atherosclerotic plaques than those reconstituted with wild-type bone marrow [8]. However, the critical role of the NLRP3 inflammasome in atherosclerosis has been challenged by another study of double-mutant mice generated by crossing* Apoe* ^−/−^ mice with* Nlrp*3^−/−^,* Pycard* ^−/−^, or* Caspase*1^−/−^ mice [10]. That study did not find any differences in atherosclerosis progression with or without the NLRP3 inflammasome. To clarify these differences, we designed this study, applying RNA interference. Our results demonstrated that silence of NLRP3 suppressed atherosclerosis and stabilized atherosclerotic plaques, as the NLRP3i group of mice consistently exhibited a lower content of macrophages and lipids, a higher content of collagen, and a thicker fibrous cap in atherosclerotic plaques than the control group of mice. All these morphological changes led to a decreased plaque vulnerability index in the NLRP3i group compared to the control group. By incorporating the cumulative effects of protective and destructive factors, plaque vulnerability index has been recognized as a histological marker for vulnerable plaques. These findings suggested that NLRP3 was critical in the progression of atherosclerosis.

Atherosclerosis progresses through lipid accumulation within macrophages, resulting in the formation of foam cell and necrotic core. Although cholesterol lowering is a way to decrease lipid retention, increasing lipid efflux from macrophages, mostly through ABCA1 and ABCG1, has been regarded as another significant strategy to limit atherosclerosis. Overexpression of ABCA1 and ABCG1 limits atherosclerotic plaque formation, while genetic deletion of these genes promotes foam cell formation and accelerates atherosclerosis in animal models [23–26]. Our data showed that mRNA levels of ABCA1 and ABCG1 were increased after the treatment of NLRP3i, suggesting that NLRP3 gene silencing could be a potentially therapeutic mean to increase macrophage cholesterol efflux for the prevention of atherosclerosis.

Collagens are the main components of fibrous cap in atherosclerotic plaques and play a vital role in stabilizing plaques and preventing plaque rupture. Prolyl-4-hydroxylase (P4H) is one of the key enzymes essential for the collagen synthesis. P4H*α*1, an isoenzyme of P4H, is a rate-limiting enzyme that folds the procollagen polypeptide chains into the stable triple helical molecules, which is necessary for collagen maturation and secretion [27]. In the present study, we showed that the mRNA expression of P4H*α*1 was significantly higher, and the mRNA expression of MMP-2 and MMP-9 that contributed to collagen degradation was markedly lower in the NRLP3 interfering group than in the control group, resulting in the thickness of fibrous cap and the stabilizing of atherosclerotic plaques.

In conclusion, the present study demonstrated that lentivirus-mediated RNA interference was effective in knocking down NLRP3 genes in ApoE deficient mice, resulting in reduced inflammatory cytokines and plaque content of lipid and macrophages, increased plaque content of collagen, and lowered plaque vulnerability index. NLRP3 inflammasomes play important roles in the pathogenesis of plaque vulnerability. Our findings raised the possibility that lentivirus-mediated gene silencing of NLRP3 is a potential therapeutic target for inhibiting progression of atherosclerotic plaques and subsequently preventing cardiovascular events.

## Figures and Tables

**Table 1:** Primer sequences for real-time PCR.

Gene	Forward sequence	Reverse sequence
NLRP3	5′-ATGCCAGGAAGACAGCATTG-3′	5′-TCATCGAAGCCGTCCATGAG-3′
ABCA1	5′-GCGGACCTCCTGTGGTGTT-3′	5′-CAAGAATCTCCGGGCTTTAGG-3′
ABCG1	5′-AAGGCCTACTACCTGGCAAAGA-3′	5′-GCAGTAGGCCACAGGGAACA-3′
P4H*α*1	5′-CCCTAAGGCAACTGGATGAA-3′	5′-CCCATTTGTTGCCAACTAG-3′
MMP-2	5′-ACCCAGATGTGGCCAACTAC-3′	5′-TCATTTTAAGGCCCGAGCAA-3′
MMP-9	5′-CCTGGAACTCACACGACATCTTC-3′	5′-TGGAAACTCACACGCCAGAA-3′
*β*-actin	5′-TCATCACTATTGGCAACGAGC-3′	5′-AACAGTCCGCCTAGAAGCAC-3′

**Table 2:** Body weight and serum lipid levels in control group and NLRP3i group.

End point	Control (*n* = 20)	NLRP3i (*n* = 20)	*P* value
Body weight, g	28.8 ± 4.1	29.1 ± 3.8	NS
Total cholesterol, mmol/L	2.07 ± 0.87	2.01 ± 0.64	NS
Triglycerides, mg/dL	35.2 ± 3.24	33.5 ± 2.4	NS
LDL, mg/dL	6.15 ± 1.48	5.89 ± 0.73	NS
HDL, mg/dL	10.35 ± 1.75	11.54 ± 1.79	NS

NS indicates not significant.
